# A Systematic Review of Common and Brain-Disease-Specific RNA Editing Alterations Providing Novel Insights into Neurological and Neurodegenerative Disease Manifestations

**DOI:** 10.3390/biom12030465

**Published:** 2022-03-17

**Authors:** Korina Karagianni, Spyros Pettas, Georgia Christoforidou, Eirini Kanata, Nikolaos Bekas, Konstantinos Xanthopoulos, Dimitra Dafou, Theodoros Sklaviadis

**Affiliations:** 1 Department of Genetics, Development, and Molecular Biology, School of Biology, Aristotle University of Thessaloniki, 541 24 Thessaloniki, Greece; korinagk@bio.auth.gr (K.K.); spyrospg@bio.auth.gr (S.P.); ge0067ch-s@student.lu.se (G.C.); mpekasns@bio.auth.gr (N.B.); 2 Neurodegenerative Diseases Research Group, Department of Pharmacy, School of Health Sciences, Aristotle University of Thessaloniki, 541 24 Thessaloniki, Greece; ekanata@bio.auth.gr (E.K.); xantho@pharm.auth.gr (K.X.); sklaviad@pharm.auth.gr (T.S.)

**Keywords:** RNA editing, brain disorders, neurodegenerative diseases

## Abstract

RNA editing contributes to transcriptome diversification through RNA modifications in relation to genome-encoded information (RNA–DNA differences, RDDs). The deamination of Adenosine (A) to Inosine (I) or Cytidine (C) to Uridine (U) is the most common type of mammalian RNA editing. It occurs as a nuclear co- and/or post-transcriptional event catalyzed by ADARs (Adenosine deaminases acting on RNA) and APOBECs (apolipoprotein B mRNA editing enzyme catalytic polypeptide-like genes). RNA editing may modify the structure, stability, and processing of a transcript. This review focuses on RNA editing in psychiatric, neurological, neurodegenerative (NDs), and autoimmune brain disorders in humans and rodent models. We discuss targeted studies that focus on RNA editing in specific neuron-enriched transcripts with well-established functions in neuronal activity, and transcriptome-wide studies, enabled by recent technological advances. We provide comparative editome analyses between human disease and corresponding animal models. Data suggest RNA editing to be an emerging mechanism in disease development, displaying common and disease-specific patterns. Commonly edited RNAs represent potential disease-associated targets for therapeutic and diagnostic values. Currently available data are primarily descriptive, calling for additional research to expand global editing profiles and to provide disease mechanistic insights. The potential use of RNA editing events as disease biomarkers and available tools for RNA editing identification, classification, ranking, and functional characterization that are being developed will enable comprehensive analyses for a better understanding of disease(s) pathogenesis and potential cures.

## 1. Introduction

RNA editing refers to an epigenetic mechanism that contributes to transcriptome diversification through the introduction of alterations in RNA species relative to the corresponding genome-encoded information (RNA–DNA differences, RDDs) [1].

**Figure 1:**
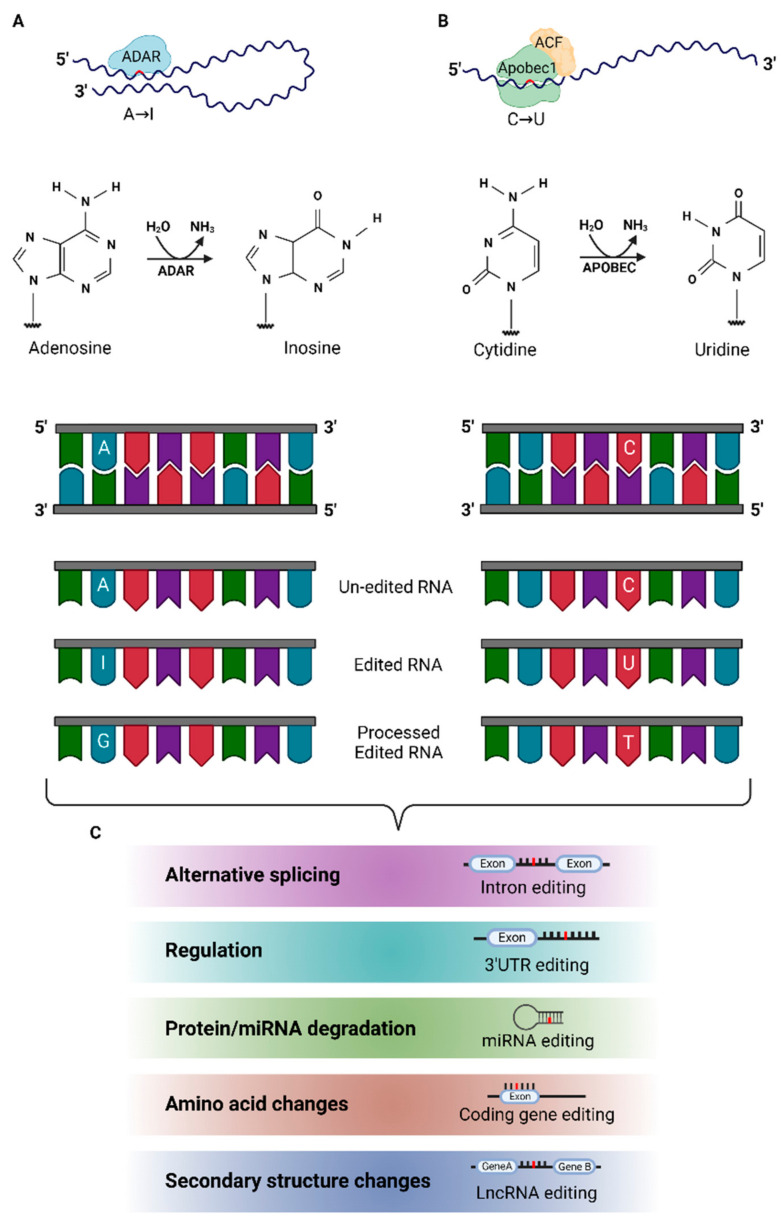
ADAR and APOBEC enzyme members catalyze RNA editing in mammals leading to epi-transcriptomic alterations. (**A**) The mammalian ADAR family comprises three members: ADAR1, ADAR2 and ADAR3. The first two are catalytically active and widely expressed; ADAR3 is expressed exclusively in the brain, has no proven catalytic activity, and is suggested to act as an ADAR1 and ADAR2 regulator. ADARs catalyze A-I editing in the form of homo- and/or heterodimers, without the requirement of other co-factors. Complementary or partially double-stranded RNAs may be used as ADARs’ substrates. Any dsRNA ≥ 20 bp, including protein-coding exons in pre-mRNAs, repetitive sequence elements, as well as microRNA (miRNA) precursor transcripts, may be ADAR substrates. ADARs deaminate Adenosine (A) to Inosine (I). The cellular transcriptional and translational machinery recognizes Inosine (I) as Guanine (G); thus, processed ADAR edited transcripts display a G at the edited site (A-I-G editing). (**B**) APOBEC1 is the main C-U editing enzyme in mammals. APOBEC1-mediated editing is highly specific and requires the formation of the editosome, a protein complex that comprises an enzyme homodimer, an essential co-factor (A1CF or RBM47) and auxiliary proteins that regulate enzymatic activity. APOBEC1 targets are ssRNAs and display specific sequence elements, corresponding to the mooring sequence (an 11 nt consensus sequence located downstream the C undergoing deamination, required for A1CF binding) and an AU-rich ‘efficiency region’, located upstream of the edited residue. APOBECs deaminate Cytidine (C) to Uridine (U). The cellular transcriptional and translational machinery recognizes Uridine (U) as Thymine (T); thus, processed APOBEC edited transcripts display a T at the edited site (C-U-T editing). (**C**) RNA editing events may occur at several sites within a transcript and affect stability, processing, and function of the edited target. Editing in intronic regions or close to splice junction sites may cause the alternative splicing of the edited transcripts. RNA editing events within non-coding regions (5′UTR, 3′UTR) may affect transcript stability and regulation. Editing events within a miRNA seed sequence may re-direct miRNA targeting and cause degradation. Introduction of non-synonymous changes within a coding region of a transcript results in amino acid alterations (protein recoding, stop codon introduction) and may also affect a transcript’s secondary structure and alter its interactions with RNA-binding proteins (RBPs). LncRNA editing can lead to changes in secondary structure affecting its regulatory functions. Figure created with BioRender.com (accessed on 31 January 2022).

The major type of RNA editing in mammals is the chemical modification (deamination) of Adenosine (A) to Inosine (I) or Cytidine (C) to Uridine (U), resulting in A-I or C-U substitutions. It occurs as a nuclear and cytoplasmic co- and/or post-transcriptional event and is catalyzed by the ADAR (Adenosine deaminases acting on RNA) and APOBEC (apolipoprotein B mRNA editing enzyme catalytic polypeptide-like) enzyme families [2] (Figure 1A,B).

Depending on the RNA species undergoing editing and the edited position(s) within a transcript, RNA editing may result in an altered structure, stability, and transcript processing; strong evidence suggests that RNA editing is a major epigenetic gene expression regulator, acting both independently and in conjunction with RNA interference pathways, the latter of which may also be subjected to the dependent regulation of RNA editing. Furthermore, protein recoding (altered amino acid encoding) and immature stop codon introduction may occur as a result of non-synonymous changes introduced by RNA editing in coding regions (Figure 1C).

RNA editing is highly regulated in a tissue- [3], developmental- [4], and cell-type- [5] dependent manner. It is involved in the fine-tuning of cellular responses to hormonal and nutritional stimuli [6] and to cellular environment alterations, including hypoxic conditions [2,7,8,9], suggesting a significant role in sustaining cellular homeostasis.

As a result, several studies have associated RNA editing with disease conditions. Type I neurofibromatosis is associated with a dysfunctional protein resulting from the introduction of a premature stop codon in the *NF1* mRNA due to false C-U editing [10]; random editing by both ADAR1 and ADAR2, as well as mutations in the APOBEC1 gene have been reported in several cancer cases [11] and ADAR mutations were identified as causative for the Aicardi–Goutières Syndrome (AGS), an inherited encephalopathy that affects newborn infants and results in severe mental and physical disability [12].

Among other tissues, the brain displays the highest editing levels in both mice and humans [13,14] and shows a gradual increase in both mRNA and miRNA editing during its development [15,16]. Neurons and astrocytes represent the most-edited brain cell types [17]. RNA editing is altered by neuronal activity [18]. A-I editing has been associated with the trafficking and assembly of kaniate [19] and GABA(A) receptors [20], modulation of Ca(v)1.3 channels activation [21,22] and regulation of Nova1 stability [23], a neuron-specific RNA-binding protein involved in alternative splicing [24]. Similar to other diseases, it is expected that brain disorders are associated with RNA editing alterations [25].

In this review, we focus on the role of RNA editing alterations in psychiatric, neurological, neurodegenerative (NDs) and autoimmune brain disorders in human cases and in related mouse models. We provide a comprehensive presentation of relevant data supporting RNA editing contribution in these disorders. We include studies focusing on editing events identified in selected, disease-associated targets, as well as recently published transcriptome-wide studies and discuss common editing patterns among different brain disorders. Furthermore, we attempt to identify commonly edited targets and affected cellular processes among human and mouse brain disorders, suggestive of common underlying molecular mechanisms and indicative of animal models’ utilization reliability. Subsequently, we discuss the significance of extending transcriptome-wide RNA editing studies at pre-clinical disease stages in relevant disease models, aiming to identify RNA editing alterations that may contribute to driver pathogenetic events, that could provide novel candidate intervention targets. Finally, we discuss future perspectives and challenges in the RNA editing study field, focusing on CNS disorders.

## 2. Materials and Methods

### 2.1. Literature Search Strategy

Five independent reviewers conducted a systematic literature search on PubMed for suitable research published between 1995 and 2021, following preset methods and intending to identify studies related to RNA editing in brain disorders. The search terms were: “RNA editing” OR “RNA alterations” AND “neurological disorders” OR “neurodegenerative diseases” OR “neurodegenerative disorders” OR “neurodegeneration” OR “psychiatric disorders”.

### 2.2. Eligibility Criteria

Studies were considered eligible if they met the following requirements: (1) Studies in humans and rodent models examining RNA editing events in relation to psychiatric, neurological, neurodegenerative, and autoimmune brain disorders; (2) genetically determined neurological and neurodegenerative disorders, influenced by environmental factors or caused by an injury; (3) studies in brain and/or spinal cord tissue material from either postmortem human material or animal disease models.

Exclusion criteria were as follows: (1) non-original publications, such as letters to the editor, opinions, reviews, case reports, protocols, conference or meeting abstracts, comments, or meta-analyses; (2) studies unrelated to the subject; (3) studies with insufficient or unqualified data.

### 2.3. Statistical Analysis and Data Extraction

Only articles written in English were considered. In addition, duplicate publications were excluded. The following information was gathered from all the studies that were included: basic information (first author, publication year, and research country), disorder, species, brain area, study type, targets, methodology, validation method, differentially expressed targets compared to controls.

### 2.4. Registration for Studies

The studies were evaluated using the criteria specified by the PRISMA (preferential reporting items for systematic review and meta-analysis) guidelines [26].

## 3. Results

The term ‘Neurological Disorder’ refers to a wide range of disorders concerning the nervous system, which are characterized by a variety of symptoms ranging from mild to severe and are either accompanied by neurodegeneration or not. Neurological disorders may be either inherited with genetic predisposition, associated with environmental factors or may occur as a result of injury. Neurological/neurodegenerative disorders, such as epilepsy, autism, Alzheimer’s disease (AD), Parkinson’s disease (PD), Huntington’s disease (HD), prion diseases, amyotrophic lateral sclerosis (ALS) and multiple sclerosis (MS), can be clustered together because they all involve the malfunction or damaging of the brain, spinal cord, and nerves. Nervous system degeneration affects neuronal communication resulting in behavior and emotional state disruptions, which also represent hallmarks of psychiatric disorders, such as schizophrenia (SCZ), chronic social defeat stress (CSDS), depression and suicide. Thus, despite their difference, neurological/neurodegenerative and psychiatric disorders display some degree of overlapping aspects and possibly common or similar pathogenetic mechanisms.

These disorders have long attracted the interest of the scientific community due to a dramatic increase in cases and their economical as well as social impact. Studies on the effect of RNA editing within these disorders’ contexts first began in the 1990s. Table 1 and Table 2 provide a summary of relevant studies conducted in brain and/or spinal cord post-mortem human autopsy or from experimental disease animal models.

As shown in Table 1 and Table 2, most of these studies focused on the analysis of selected RNA editing events, frequently resulting in the recoding of neuron-enriched transcripts with established roles in neuronal function. Indeed, studied targets included A-I-edited transcripts encoding neurotransmitter receptors, such as the AMPA and kainate ionotropic glutamate receptor subunits encoded by the *Gria2*, *Gria3* and *Gria4* and by the *Grik1* and *Grik2* transcripts, respectively; the serotonin receptor, encoded by the 5-HT2C transcript; and the *KCNA1* encoded alpha subunit of the Kv1.1 potassium channel, which all have established roles in neuronal excitability [15].

Among these, the *Gria2* encoded subunit of the AMPA glutamate receptor seems to be the most significant, as the introduction of the Q/R recoding site in ADAR2-deficient mice prevents animal lethality [58], which occurs as a result of neurotoxicity due to increased Ca^++^ permeability. Given the prominent role of this recoding event, it is not surprising that *Gria2* was selected as a study target in the context of several brain disorders, including HD, AD, ALS, SCZ and has been detected as under-edited in these disease conditions (Table 1 and Table 2).

Indeed, several studies indicated that reduced RNA editing at the GluA2 Q/R site increases the influx of Ca^++^, leading to excitotoxicity, and a negative effect on motor neurons in ALS [59]. Increased Ca^++^ influx leads to calpain activation. Calpain acts as a protease, cleaving TDP-43 into aggregation-prone fragments. These fragments accumulate progressively due to the constant calpain activation caused by elevated intracellular levels of Ca^++^, resulting in the mislocalization and aggregation of TDP-43 in the cytoplasm, a key characteristic of ALS [60,61]. This is also confirmed by a study with ALS patients, in which all ADAR2-deficient motor neurons demonstrated TDP-43 mislocalization [62].

Moreover, it was demonstrated that RNA editing plays a pivotal role in Ca^++^ permeability of AMPA receptor ion channels in epilepsy and it was suggested that GluA2 Q/R RNA editing is associated with seizure susceptibility [58,63]. Highly increased levels of intracellular Ca^++^ can be lethal, leading to fatal epilepsy in a mouse model expressing the unedited GluA2 [63]. ADAR2 deficiency also appears to induce seizures during the early postnatal stages in mice [58]. Additionally, conditional mouse mutants, in which GluA2 editing was inactivated postnatally in selected forebrain regions, showed synaptic changes implying a susceptibility to seizure manifestation [64]. This suggests that the epileptic phenotype of mouse models with GluA2 Q/R-editing deficits may be a direct consequence of altered AMPA receptor properties in the adult brain.

In addition, RNA editing events at the R/G site of *Gria2*, *3* and *4* transcripts have also been shown to modulate the speed of inactivation following depolarization. It has been suggested that reduced editing at the *Gria2* R/G site may compensate for glutamate over-stimulation, thus providing a compensatory and protective effect against neuronal death. On the other hand, increased editing at this site, reported in the epileptic hippocampus [29], was suggested to result in the overactivation of the receptor and to promote chronic Ca^++^ overload, thus contributing to the progression of epilepsy.

Apart from the connection of RNA editing at the *Gria2*-encoded subunit of the AMPA glutamate receptor with several brain disorders, the GluK1 and GluK2 subunits of kainite receptors (KA) also possess the Q/R RNA editing site, which also affects Ca^++^ permeability. Therefore, it was suggested that altered RNA editing in these sites might also contribute to epileptogenesis. KA-induced epilepsy leads to increased GluK1 Q/R site editing following seizures in the hippocampus in a rat model [65]. Additionally, GluK1 and GluK2 Q/R RNA editing was found to be elevated in the hippocampus and temporal cortex of epileptic patients [28]. In addition, increased GluK2 Q/R RNA editing was accompanied with ADAR2 upregulation in the hippocampus of patients with refractory epilepsy [27].

Another RNA editing event that seems to be connected to epileptogenesis is the I/V site of the potassium channel Kv1.1. This ADAR2 catalyzed isoleucine (I) to valine (V) editing takes place in the pore domain of the channel, resulting in a rapid recovery from fast inactivation. This editing event increases fourfold in the entorhinal cortex of chronic epileptic rats compared to their healthy counterparts [64]. Mutations in the gene that encodes Kv1.1 (KCNA1) that hinder the Kv1.1 I/V site editing are also hypothesized to be responsible for the epileptic events observed in patients suffering from episodic ataxia type 1 (EA1) [66].

Moreover, given that abnormalities in serotonergic transmission are strongly associated with mood and psychiatric disorders, it is plausible that the serotonin receptor *5-HT2C* transcript has been selected as a study target in depression and suicide studies, as well as in autism, SCZ and AD. The *5-HT2C* transcript is known to undergo A-I editing at five sites (A–E) within the region involved in G-protein coupling. Editing reduces the receptors’ G-protein coupling and thus its function. The differential representation of *5-HT2C* isoforms resulting from the combination of editing at positions A-E was reported in these disorders and associated with disease progression. Additionally, reduced editing reported in the *KCNA1* transcript in epilepsy was inversely correlated with disease duration [30].

Apart from neuronal transcripts, a few studies have focused on glial targets. *PDE8A* is expressed mainly in oligodendrocytes (Brain RNA-seq, https://www.brainrnaseq.org/, (accessed on 31 January 2022)) and encodes a phosphodiesterase involved in signal transduction affecting inflammatory responses, memory and cognition. *PDE8A* was studied in terms of RNA editing alterations in depression and suicide. Similar to *5-HT2C*, several editing sites were reported in *PDE8A* and differential representation of the corresponding edited transcripts was detected in depression and suicide, providing novel targets for further functional characterization [42]. Editing alterations have also been reported in *EAAT*, another glia-enriched transcript, expressed mainly in astrocytes. This editing alteration, detected in AD, occurs in intron 7 and affects intron retention, as shown by in vitro functional evidence [48].

The previously mentioned, strictly focused, studies were extended by studies focusing on transcripts involved in known disease-related molecular pathways [43,44,53], providing better insights into the contribution of RNA editing in central nervous system (CNS) diseases.

Advances in high-throughput transcriptomic studies, achieved through the development of efficient experimental technologies and robust bioinformatics approaches for data analysis, significantly contributed to RNA editing research. Indeed, several ADAR- and APOBEC-mediated editing events were reported in different organisms, and corresponding databases (RADAR, rigorously annotated database of A-to-I RNA editing [67], DARNED [68], and REDIportal [69] have been created).

These studies have shown that, among the thousands of RNA editing events identified, only a small proportion was found within coding regions, and an even smaller number of them resulted in non-synonumous changes; in contrast, the majority of editing events is detected in non-coding regions, such as intergenic and intronic regions [70], 3′ UTRs [13,69,70,71,72,73,74] and 5′ UTRs, where repetitive elements such as the Alu/SINE elements are found [75]. This observation is particularly interesting, as it suggests functional roles of RNA editing within non-coding regions and allows for the extension of relevant studies beyond the handful of non-synonymous, recoding events studied so far. This is expected to contribute to the identification of novel disease-associated targets, with extensions to disease pathogenesis, diagnosis and/or therapeutics.

Transcriptome-wide analyses allow for the unbiased identification of disease-related changes and were recently employed for RNA editing studies in health and disease conditions. As of now, few transcriptome-wide studies on RNA editing alterations within the context of neurodegenerative/neurological and psychiatric disorders have been conducted. These include human diseases (autism [45], schizophrenia [35], genetic-C9orf72 ALS [51], Alzheimer’s disease [55]), in vivo models of epilepsy [31], and prion diseases [57]. Interestingly, in most of these studies, reduced global editing was associated with disease, highlighting a ‘protective’ role of RNA editing against disease progression; most importantly, these studies will allow for the identification and subsequent functional characterization of novel disease-associated targets.

A link between RNA hypoediting and the neurodevelopmental condition of autism has been recently revealed through a transcriptome-wide study [45]. The authors highlighted RNA editing downregulation in transcripts involved in synaptic functions and in targets corresponding to ASD susceptibility genes. Furthermore, they identified a small number of ASD-related transcripts displaying concurrent and differential editing and gene expression alterations (e.g., *KCND2*, *GRIK2*), thus providing some functional molecular links between RNA editing alterations and ASD. Interestingly, significantly overlapped patterns of editome alterations were observed in autism spectrum disorder (ASD) and fragile X syndrome (FXS), suggesting RNA editing as a link between ASD and FXS. FXS is caused by the silencing (methylation) of the FMR1 gene that is often present in features of ASD and accounts for about 5% of ASD cases [76].

Another transcriptome-wide analysis aimed to identify differentially edited sites in the hippocampus of a mouse model with temporal lobe epilepsy relative to healthy controls [31]. The authors identified 256 differentially edited sites, residing in 87 transcripts, including epilepsy-related targets. The differential editing in these sites was positively correlated with the occurrence of seizures and epilepsy. Importantly, a subset of differentially edited targets identified in the epileptic mouse model were also found to undergo RNA editing in human epileptic hippocampi, further suggesting their relevance to epilepsy [31].

Finally, a systematic transcriptome-wide evaluation in a large dataset from the human cortex and cerebellum has uncovered a significant connection between RNA editing and Alzheimer’s disease. Through a correlation analysis and RNA editing ranking, the study highlights several RNA editing events highly associated with AD (e.g., mitochondria-associated targets, including *SOD2*, *MCUR1*, *PFKP* and transcripts involved in fatty acid and lipid metabolism, such as *HSDL2*). In addition, by utilizing multi-omics (transcriptomics, proteomics and neuropathological traits) integration approaches, the authors highlight a negative correlation of RNA editing in the 3′UTR of *SYT11* with SYT11 protein levels, and a correlation of RNA editing at the 3′-UTR of *ORAI2* with aggregation of hyperphosphorylated tau and with neuritic amyloid plaque burden, suggesting functional molecular links between RNA editing alterations and AD [55].

**Figure 2:**
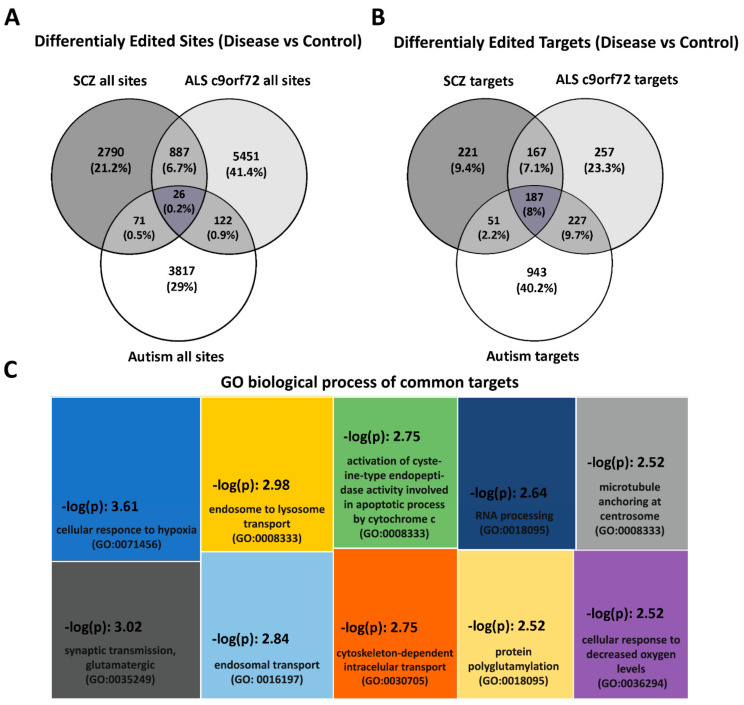
Differential editing patterns in different human neuropsychiatric, autoimmune/neurodegenerative and neurological brain disorders compared to healthy individuals. (**A**) Venn diagrams depicting differentially edited sites identified in schizophrenia (SCZ), genetic ALS (ALS c9orf72) and autism. The intersections represent editing events identified as differentially edited in more than one condition. Twenty-six editing sites have been commonly identified as differentially edited in all three conditions. (**Β**) Venn diagrams depict differentially edited transcripts (targets) in the same human disorders as in A. The intersections represent editing targets identified as differentially edited in more than one condition. Please note that a higher number of transcripts (187) are differentially edited in all three conditions compared to common, individual editing events (intersection in A), suggesting disease-associated editing that converges in common targets. (**C**) Gene ontology (GO) analysis of the differentially edited targets in all three human brain disorders (*n* = 187). Enrichment values are given as –log (*p*) values (higher –log (p) values indicate greater statistical significance). Differentially edited transcripts are involved in molecular processes associated with hypoxia, synaptic transmission, endosome/lysosome function, cytoskeleton, apoptosis, protein and RNA processing, all of which have been reported as deregulated in these disorders. Differential editing data (*p* < 0.05) reported in the studies of [35,45,51] were utilized for the analysis. Reported data correspond to different brain regions.

Remarkably, a comparative analysis of these studies’ data unraveled common, differentially edited sites and transcripts among different human brain disorders (Figure 2). Indeed, 26 editing events (chr1:155853123, chr1:160112452, chr1:86119319, chr10:15119402, chr10:82192352, chr12:121089976, chr12:51324192, chr12:72096123, chr14:101322726, chr14:74525531, chr15:55668134, chr15:90618580, chr16:75479250, chr19:23542087, chr19:59095532, chr2:206986949, chr22:24968618, chr3:14986471, chr3:169807487, chr3:179115692, chr5:150647846, chr7:130629894, chr8:38829925, chr9:115233907, chrX:73421922, and chrX:73445571; coordinates given relative to hg19 Reference Genome) are commonly reported as being differentially edited relative to corresponding controls in schizophrenia, genetic ALS (C9orf72) and autism (Figure 2A). Interestingly, when the same comparison entailed differentially edited transcripts, a 7-fold increase in common differentially edited targets (187) was observed (Figure 2B). These data suggest that different brain disorders display both common (common sites and targets) and disease-specific (individual) RNA editing signatures, possibly involved in disease-associated molecular processes. In support to this notion, a pathway analysis identified the enrichment of these common targets in molecular processes associated with synaptic transmission, the response to hypoxic conditions, endosome/lysosome transport, apoptosis and cytoskeleton rearrangements (Figure 2C), commonly occurring in these disease conditions [77,78,79,80,81].

Despite being highly informative, studies of human postmortem CNS tissue are hampered due to limited availability of autopsy material. Studies of in vivo animal disease models may compensate for this limitation. However, animal disease models usually fail to fully recapitulate the whole range of human disease aspects. This is relevant in RNA editing studies, as species-specific differences may affect editing enzymes targeting and function, thus stressing that a direct extrapolation of mouse-to-human RNA editing events may not always be possible. However, if RNA editing has functional effects on disease progression, differentially edited transcripts would at least partially overlap and, most importantly, converge to common disease-associated pathways between human and mice.

**Figure 3:**
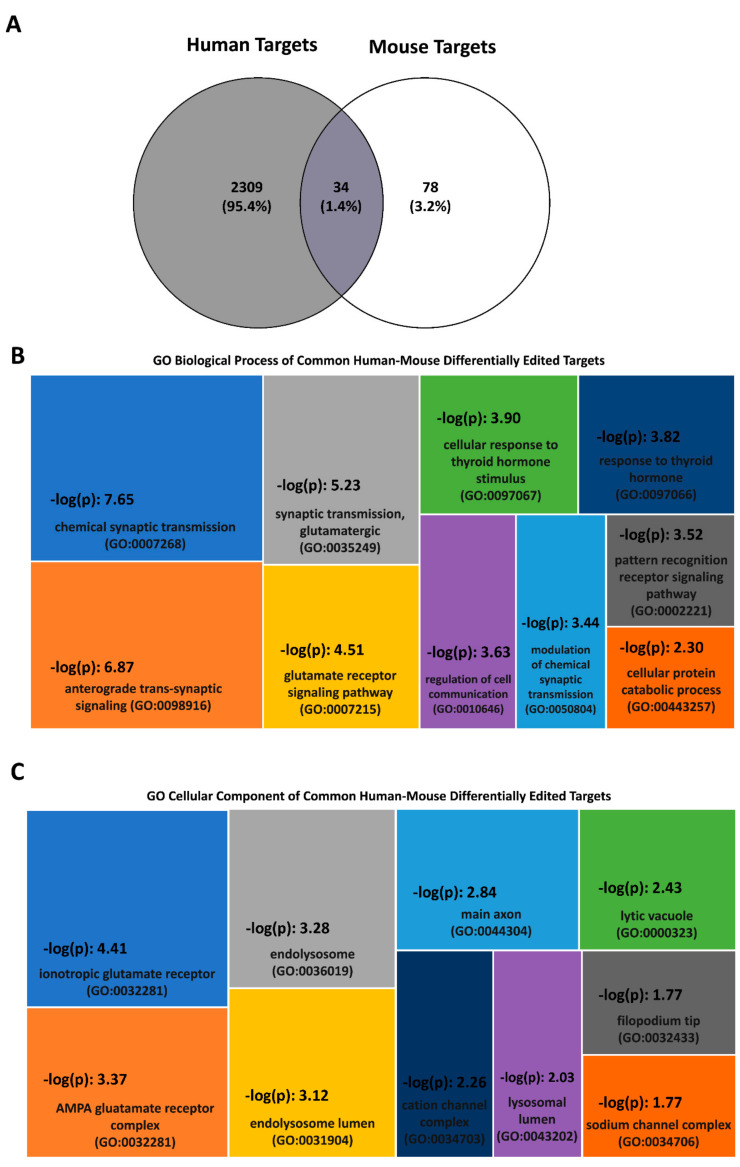
Differentially edited targets in human neuropsychiatric, autoimmune/neurodegenerative, neurological disorders [35,45,51] and in neurological/neurodegenerative disease mouse models [31,43,57] compared to healthy controls. (**A**) Venn diagram depicting differentially edited transcripts identified in humans and mice compared to healthy controls. The intersection represents editing targets commonly identified as DE in human and mouse brain disorders. (**B**,**C**) Gene ontology (GO) analysis of the differentially edited targets in human and mouse brain disorders (*n* = 34). Enrichment values are given as –log (p) values (higher –log (*p*) values indicate greater statistical significance). (**B**) Biological process enrichment. Common DE transcripts are involved in molecular processes associated with synaptic transmission and signaling, regulation of cell communication, pattern recognition receptor signaling, response to thyroid signaling and protein catabolism. (**C**) Cellular component enrichment. Main axon, glutamate receptor, cation and sodium channel complexes, lytic vacuoles and specifically endo/lysosome lumen, as well as filipodium tip, emerge as cellular component terms enriched in DE transcripts in both human and mouse brain disorders.

Indeed, a comparative analysis of differentially edited targets detected in at least one of the human and mouse neurological/neurodegenerative and psychiatric disorders studied (data obtained from [31,35,43,51,57]) identified overlaps between species (Figure 3A). Specifically, 34 transcripts were reported as similarly differentially edited in at least one of the studied disorders in both human and mice (*Abi2*, *Agbl4*, *Ankrd28*, *Arf3*, *Bri3bp*, *Cds2*, *Copa*, *Cpe*, *Ctsb*, *Ctss*, *Dnajc18*, *Fam107a*, *Gabra3*, *Gnl3l*, *Gria2*, *Gria4*, *Grik2*, *Hspa4l*, *Itm2b*, *Mbp*, *Mdga2*, *Meg3*, *Nova1*, *Nup155*, *Paqr8*, *Rragd*, *Samd8*, *Scn1b*, *Slc1a2*, *Slc35e1*, *Smim14*, *Sparcl1*, *Spock2*, *Tcp11l1).* Interestingly, these targets are enriched in biological processes related to synaptic transmission and signaling, the regulation of cell communication, pattern recognition receptor signaling, response to thyroid signaling and protein catabolism (Figure 3B), thus confirming the involvement of RNA editing in common disease-related processes. Synaptic processes, representing the major molecular hallmark of CNS disorders, are the most (affected) represented functions. Moreover, a cellular component analysis revealed enrichment in the terms ‘main axon’, ‘glutamate receptor’, ‘cation’, ‘sodium channel complexes’, ‘lytic vacuoles’, ‘endo/lysosome lumen’, and ‘filipodium tip’, thus providing more detailed insights into RNA editing contribution to disease (Figure 3C).

These data highlight common disease-related processes in human and mice and support the utility of RNA editing studies in murine disease models, provided that species-specific differences possibly affecting editing patterns. However, an experimental cross-validation of novel differentially edited targets should be performed in human autopsy tissue.

Of note, all of the above processes, determined at end-point (human) or significantly advanced (mice) disease stages, possibly reflect common end-stage disease consequences rather than disease promoting events. When similar studies would be conducted at earlier disease stages will significantly aid towards the identification of disease driver/promoting RNA editing events. Thus, pre-clinical studies utilizing animal models are highly needed. To our knowledge, there is currently only one published study reporting pre-clinical disease alterations of RNA editing patterns [57]. This study reported differentially edited transcripts potentially involved in early synaptic dysfunction in Prion diseases.

## 4. Discussion

### 4.1. The Diagnostic/Prognostic Potential of RNA Editing in Neurological/Neurodegenerative and Psychiatric Disorders

Altered RNA editing profiles within *Alu* regions in the brain of individuals with glioblastoma were suggested as a prognostic factor in a gender-dependent manner and decreases in global A-I editing profiles were reported as an efficient patient stratification approach in this disease [82]. Moreover, the loss of editing in GABRA3 was associated with an aggressive phenotype of glioma, promoting migration and invasion [82].

Peripheral blood analysis from healthy individuals, detected more than 1000 RNA editing sites [83]. Moreover, increased A-I editing within the *AluSx+* sequence, which is in the *Ctss* 3′UTR and affects *Ctss* expression, was reported in peripheral blood mononuclear cells of rheumatoid arthritis (RA) patients [84]. The same authors also identified altered editing as a response to drug treatment [84]. Furthermore, a recent review discusses the possibility of exploiting RNA editing biomarkers for drug development against suicidal ideation [85].

The above data suggest that RNA editing profile studies in body fluids may enhance disease diagnosis as well as patient responses to treatments. Indeed, a recent study reports characteristic *PDE8A* RNA editing patterns in the blood of depressed patients and suicide attempters with major depression, suggesting its diagnostic potential [86]. In addition, a recent review of biomarkers for ALS and other NDs suggested altered RNA editing efficiencies in ADAR2-dependent sites as candidate biomarkers [87]. Furthermore, a recent transcriptome-wide RNA editing analysis performed on peripheral blood from a mutli-ethnic group of AD patients and corresponding control samples, identified differentially edited targets converging in endocytic and inflammatory pathways [88]. This further supports the diagnostic potential of RNA editing studies of body fluids. However, even though RNA editing may hold a promising diagnostic and prognostic potential, additional studies are required.

### 4.2. Future Perspectives and Challenges in the RNA Editing Research Field

The existing literature indicates RNA editing as an emerging mechanism that contributes to disease progression and highlights common and disease-specific editing patterns.

The advent of transcriptome-wide studies has enabled a comprehensive profiling of RNA editing alterations in health and disease conditions. Further studies focusing on different disorders are expected to extend the so-far-limited data on transcriptome-wide and descriptive studies of RNA editing. A significant challenge in this field refers to the accurate identification of true RNA editing events, which is based on comparative analyses of RNA-seq data aligned against the corresponding reference genome for the detection of RNA:DNA differences (RDDs). Both experimental setup and data analysis pipelines affect the accuracy and efficiency of identifying RNA editing events. Accurate alignment in the reference genome was identified as a critical step influencing the correct calling of RNA editing, and several alignment approaches were reported (some of them reviewed in [89]). False-positive RDDs detection is an inherent pitfall of RNA editing analysis approaches, and extensive research has aimed to develop improved data analysis pipelines that incorporate several filtering steps to limit false discovery rates. These filtering steps refer to stringent quality-control criteria of base quality and read depth; the exclusion of reads mapping to multiple genomic loci, pseudogenes and known SNPs; and the application of statistical analyses to evaluate editing calling efficiency and estimate false discovery rates. VarScan [90], RediTools [14,91], GIREMI [92], SPRINT [93], JACUSA [94] and RNAEditor [95] are some of the currently available RNA editing calling tools, of which the efficiency of some has recently been evaluated using both simulated and real data sets [89].

Importantly, and considering that RNA editing is tissue- and cell-type-specific, it is plausible to assume that refined studies focusing on specific cell sub-types or even on single-cell analyses, will help to unravel cell-type-specific, disease-related differences that may be undetectable in bulk tissue analyses. In this direction, few recent studies have reported cell-type-specific editing patterns in the human brain [96], murine macrophages and dendritic cells [73].

Further improvements of existing pipelines to allow for a more efficient analysis of editing events residing in long non-coding RNAs [97], and the development of algorithms for identification of RNA editing in miRNAs [98], have also been reported. Following accurate RNA editing identification, powerful statistical analysis approaches are required for the classification and ranking of RNA editing events, and for the identification of affected pathways and networks, which would allow for the selection of the most potent editing events for further functional analyses. Integrative approaches entailing multi-omics, neuropathological and cognitive status data significantly contribute to this direction. In addition, robust predictions of the functional effects of RNA editing on gene expression, protein recoding, miRNA binding and alternative splicing is highly desired. Advanced bioinformatics tools for such functional predictions applied to large-scale data, as recently reported for AD [99], will significantly contribute towards this direction.

Functional studies to delineate factors leading to altered editing and the estimation of RNA editing effects are also required. For the study of the functional effects of RNA editing, appropriate in vivo (e.g., editing deficient animal models [71,73]) and in vitro systems [100] are used. Further, novel, recently developed experimental approaches allowing site-directed A-I [101,102,103] and C-U [104,105] RNA editing modification may be utilized for functional RNA editing studies; however, further optimization is required. These functional studies should also consider the additional complication of evaluating different editing-related isoforms occurring as the result of the concomitant occurrence of several editing events in proximity within a selected target. Another important aspect in the field of RNA editing in relation to disease condition(s) refers to the delineation of RNA editing modulatory mechanisms driving or resulting in the observed RNA editing aberrations. To this end, several studies have attempted to correlate RNA editing alterations with the expression levels of the main RNA-editing mediating enzymes. The increased expression of APOBEC transcripts was reported in epilectic mice compared to controls, while ADAR1 and ADAR2 were identified as downregulated [31]. In addition, a recently published study, entailing many AD cases and controls, reported reduced ADAR2 expression in AD [55]. Interestingly, the same study identified increased ADAR3 expression; ADAR3 represents a potential RNA editing inhibitor, which blocks ADAR1 or ADAR2 catalytic activity through heterodimerization with either ADAR1 or ADAR2. In contrast, no significant alterations in ADAR1 or ADAR2 expression levels were detected in autism and C9orf72 ALS [45,51]. However, other RNA editing modulatory mechanisms have been suggested in these disorders. In the autism-related study, the authors provided experimental evidence on protein–protein interactions between FMRP-ADAR1, FMRP-ADAR2 and FXR1P-ADAR1, which directly affect RNA editing at selected transcripts, thus assigning RNA editing modulatory effects to FMRP (enhancer) and FXR1P (repressor) [45]. Moreover, widespread RNA editing alterations reported in familial ALS cases were associated with ADAR2 mislocalization, as evidenced by studies in postmortem tissues of ALS patients with C9orf72 mutations, as well as in vitro and in vivo disease models [51]. These studies highlight some of the molecular mechanisms resulting in altered RNA editing in different disease contexts. By utilizing advanced bioinformatics and technological approaches, additional RNA modulatory mechanisms are expected to be discovered and validated, thus contributing to a better understanding of molecular links between altered RNA editing and disease.

## 5. Conclusions

In summary, RNA editing studies provide significant insights in terms of CNS disease-related molecular mechanisms, suggesting a protective role in disease progression, which could be exploited in subsequent diagnostic/prognostic and even therapeutic approaches. The currently available data are mostly descriptive and further studies are required to extend the global editing profiles for different CNS disorders. Comprehensive analyses through the development of more efficient tools for RNA editing identification, classification, ranking, and functional characterization are being developed and are expected to allow for a better understanding of the role of RNA editing in these disorders.

## Figures and Tables

**Table 1:** Studies of RNA editing alterations in neurological and psychiatric disorders, utilizing either human postmortem or murine mouse models central nervous system (CNS) material. DE: differentially edited, SCZ: schizophrenia, CSDS: chronic social defeat stress, HPC: hippocampus, TC: temporal cortex, FC: frontal cortex, ACC: anterior cingulate cortex, DLPFC: dorsolateral prefrontal cortex, PFC: prefrontal cortex, BLA: basolateral amygdala, CB: cerebellum, RFLPS: restriction fragment length polymorphism, NGS: next-generation sequencing, CE-SSCP: capillary electrophoresis single-stranded conformational polymorphism, DHPLC: denaturing high performance liquid chromatography, mmPCR_seq: microfluidics multiplex polymerase chain reaction and deep sequencing, ns: non-significant.

Disorder	Species/Brain Region	Study Type/Target(s)	Methodology/Validation Method	DE Targets/Trend Relative to Controls	Remarks	Ref.
Epilepsy	Human/HPC	Focused/Grik2	RFLPs	Grik2: ↑ Q/R site	Suggested as a compensatory mechanism	[27]
	Human/TC	Focused/Grik1 and Grik2	RFLPs	Grik1: ↑ Q/R site Grik2: ↑ Q/R site	Suggested as a compensatory mechanism	[28]
	Human/HPC	Focused/Gria2	RFLPs	Gria2: ↑ R/G site	Suggested as contributor in disease pathogenesis	[29]
	Human/HPC	Focused/Gria2-4 Grik1-2, KCNA1, 5-HT2C	Sanger sequencing	KCNA1: ↓ I/V site	Inversely associated with disease duration	[30]
	Mouse/HPC	Transcriptome-wide/whole transcriptome	NGS/Sanger sequencing	19 DE targets experimentally validated, *↑ Grik2*, *Ctsb*, *Rpa*, *Sparc*, *Slc1a2*, *Ovca2*, *↓ Ncl*, *Wipi2*, *Klhl24*, *Hspa4l*, *Slc37a3*, *Cyfip2*	DE targets involved in disease related pathways	[31]
SCZ	Human/PFC	Focused/Gria2	RFLPs	Gria2: ↓ Q/R site	Possible contributor to disease pathogenesis	[32]
	Human/FC	Focused/5-HT2C	Cloning and Sanger sequencing	5-HT2C: ↓ site B	↑ Unedited isoform	[33]
	Human/PFC	Focused/5-HT2C	Cloning and Sanger sequencing	5-HT2C: ns site differences	Trend: ↑ unedited isoform	[34]
	Human/ACC/DLPFC	Transcriptome-wide/whole transcriptome	NGS meta-analysis/independent (validation) cohort in silico analysis	>100 DE sites per region, DE overlap between brain regions (*n* = 29)	↑ Global editing, DE targets involved in disease associated pathways	[35]
Suicide	Human/PFC	Focused/5-HT2C	Primer extension	5-HT2C: site A	↑ Site A	[36]
	Human/PFC	Focused/5-HT2C	Targeted NGS/cloning and Sanger sequencing	5-HT2C isoforms due to editing	↑ ABCD isoform (hypoactive)	[37]
	Human/PFC	Focused/5-HT2C	Targeted NGS/validation cohort analysis	5-HT2C edited isoforms, ↑ ABCD isoform	ABCD isoform associated with gene expression alterations	[38]
	Human/ACC/DLPF	Focused/5-HT2C	CE-SSCP	5-HT2C edited isoforms, *ACC: ↑ A*, *ABDE*, *↓ D/DLPFC: ↑ AB*	Region-specific differential representation	[39]
Depression and suicide	Human/PFC	Focused/5-HT2C	Cloning and Sanger sequencing	5-HT2C: ↑ site C’ (Ε), ↓ site D		[40]
	Human/PFC	Focused/5-HT2C	Primer extension and DHPLC/Sanger sequencing	5-HT2C: sites A and D ns	Trend, Depr.: ↑ D, Suicide: ↑ A	[41]
	Human/ACC/DLPFC	Focused/PDE8A	CE-SSCP	PDE8A edited isoforms, *ACC: ↑ ABCEF*, *ABC*, *ABEFG*, *BFG ↓ B*, *ABDE/DLPFC: ↑ ABEFG*, *BCEG*, *↓ ABF*, *BEG*	Region specific differential representation	[42]
	Human/whole blood	Focused/PDE8A	Targeted NGS	PDE8A: ↓ sites B, C, E and D, F ns ↓ Isoforms B, BC, BD, BE, BF	Similar patterns with the brain of suicide decedents	[42]
CSDS	Mouse/PFC/BLA	Focused/recoding in neuronal function related transcripts (551 sites)	Targeted NGS (mmPCR_seq)	*PFC: ↑ Commd2*, *Rsad1*, *Iqgap1*, *Klf16*, *Nova1 ↓ Wipi2*, *Zfp81*, *Rn45s*, *Rwdd2b*, *Dagla*, *BLA: ↑ Htr2c (C*, *D site)*, *Gabra3*, *Tcp11l1*, *Qpctl ↓ Zfp324*, *Copa*, *Gria4*, *Fubp3*, *Nova1*	Region-specific DE	[43]
Autism	Human/CB	Focused/synaptic transcripts (10 targets)	Pyrosequencing/validation: Padlock probes and NGS (5 targets)	Gria 4: ↑ R/G site, Grik2 and 5-HT2C edited isoforms differential representation	Gria4 editing associated with differential splicing isoform usage	[44]
	Human/TC, FC, CB	Transcriptome-wide/whole transcriptome	NGS/2nd cohort meta-analysis/cloning and Sanger sequencing	*↑ Ctsb*, *Neat1 ↓ Gsk3b*, *Nova1*, *Grik1*, *FAM213A*, *Dennd3*	↓ Global editing	[45]

↓ denotes reduced levels of editing, ↑ denotes increased levels of editing.

**Table 2:** Studies on RNA editing alterations in neurodegenerative disorders utilizing either human postmortem or murine mouse models central nervous system (CNS) material. DE: differentially edited, ALS: amyotrophic lateral sclerosis, HD: Huntington’s disease, AD: Alzheimer’s disease, vCJD: variant Creutzfeldt–Jakob disease, sCJD: sporadic Creutzfeldt–Jakob disease, SC: spinal cord, FC: frontal cortex, CB: cerebellum, ACC: anterior cingulate cortex, DLPFC: dorsolateral prefrontal cortex, PCC: posterior cingulate cortex, aPFC: anterior prefrontal cortex, pSTG: posterior superior temporal gyrus, IFGo: pars opercularis of the inferior frontal gyrus, FFG: fusiform gyrus, TC: temporal cortex, PFC: prefrontal cortex, HPC: hippocampus, RFLPS: restriction fragment length polymorphism, NGS: next-generation sequencing, hiPSC-MNs: human induced pluripotent stem cell-derived motor neurons, mmPCR_seq: microfluidics multiplex polymerase chain reaction and deep sequencing, ns: non-significant.

Disorder	Species/Brain Region	Study Type/Target(s)	Methodology/ Validation Method	DE Targets/Trend Relative to Controls	Remarks	Ref.
ALS	Human/SC	Focused/Gria2	RFLPs/Sanger sequencing	Gria2: ↓ Q/R site		[46]
	Human/neurons ^$^	Focused/Gria2	RFLPs	Gria2: ↓ Q/R site	No editing changes in Purkinje cells	[47]
	Human/SC and motor cortex	Focused/EAAT2 (astroglial glutamate transporter)	Cloning and Sanger sequencing	EAAT2: ↑ intron7	Alternative polyadenylation and intron 7 retention transcripts (in vitro functional evidence)	[48]
	Human/SC neurons ^$^	Focused/Gria2	RFLPs	Gria2: ↓ Q/R site		[49]
	Human/SC	Transcriptome-wide/focus on database listed A-I editing sites	NGS	Gria2: ↓ Q/R site ns trend	Low sample number, *n* = 5–6/group	[50]
ALS (C9orf72)	Human/SC, motor cortex, FC, CB	Transcriptome-wide/whole transcriptome	NGS/ADAR1 and/or ADAR2 deficient hiPSC-MNs cells and cells with aberrant ADAR2 localization	1526 DE transcripts	No changes in global editing, region-specific hypo- and hyper-edited patterns	[51]
HD	Human/striatum	Focused/Gria2	RFLPs	Gria2: ↓ Q/R site		[32]
AD	Human/PFC	Focused/Gria2	RFLPs	Gria2: ↓ Q/R site		[32]
	Human/HPC	Focused/Gria2	Sanger sequencing/primer extension	Gria2: ↓ Q/R site		[52]
	Human/HPC, temporal and frontal lobe	Focused/recoding in synaptic transcripts (72 targets, 118 sites)	Targeted NGS (mmPCR_seq)	↓ 5-HT2C receptor isoforms, *HPC: ↓ Cacna1d*, *Ddx58*, *Fbxl6*, *Fis1*, *Flj43663*, *Gria3*, *Gria4*, *Igfbp7*, *Kcna1*, *Meg3*, *Narf*, *Nova1*, *Ptpn14*, *Unc80 ↑ Copa Temporal lobe: ↓ Ccni*, *Fbxl6*, *Flj43663*, *Gria2*, *Gria4*, *Grik1*, *Grik2*, *Meg3*, *Mfn1*, *Tme63b*, *Unc80 ↑ Narf/Frontal lobe: ↓ Mfn1*, *Grik2*, *Meg3*, *Gria2*, *Unc80*, *Ddx58*	↓ Recoding	[53]
	Human/HPC	Transcriptome-wide	NGS	11 DE targets, *↓ Gria2*, *Gria3*, *Gria4*, *Grik1*, *Grik2 ↑ Blcap*, *Copa*, *Vn1r1*, *Znf235*, *Znf397*, *Znf582*	↓ Recoding	[54]
	Human/ACC/DLPFC/PCC/aPFC/pSTG/IFGo/FFG/CB/TC	Transcriptome-wide/focus on database listed A-I editing sites	NGS	↓ Editing in *SYT11*, *MCUR1*, *SOD2*, *ORAI2*, *HSDL2*, *PFKP*, and *GPRC5B*	DLPFC Samples: ↓ ADAR1 *↑ ADAR3 expression in AD cases*	[55]
Prion diseases	sCJD and vCJD Rhesus monkeys/CB	Focused/Alu	Cloning and Sanger sequencing	↓ Alu editing	Strain specific differences	[56]
	sCJD Mouse/Cortex	Transcriptome-wide	NGS and Sanger sequencing	3 DE targets experimentally validated, *Mouse pre-clinical: ↓ Sidt2*, *↑ Fkrp/Mouse clinical: ↑ Rragd*	↓ Global editing, *Human cross-validation: ↓ Paqr8*, *↑ Ctss*, *Rrgad*	[57]

^$^ microdissected, ↓ denotes reduced levels of editing, *↑* denotes increased levels of editing.
